# Charting the cascade of physical activities: implications for reducing sitting time and obesity in children

**DOI:** 10.1186/s44167-024-00053-9

**Published:** 2024-06-13

**Authors:** Samad Esmaeilzadeh, Pirjo Hakala, Päivi Berg, Jo Salmon, Tiina Rinne, Arto J Pesola

**Affiliations:** 1 Active Life Lab, South-Eastern Finland University of Applied Sciences, Mikkeli, Finland; 2 University of Mohaghegh Ardabili, Ardabil, Iran; 3 Juvenia – Youth Research and Development Centre, South-Eastern Finland University of Applied Sciences, Mikkeli, Finland; 4 Institute for Physical Activity and Nutrition, Deakin University, Geelong, Australia; 5 Department of Built Environment, Aalto University, Espoo, Finland

**Keywords:** Body mass index, High-intensity activity, Latent profile analysis, Light-intensity physical activities, Sitting time, Structural equation modeling

## Abstract

**Objective:**

Traditional intensity-based physical activity measures and variable-centered statistics may not fully capture the complex associations between sitting time, physical activity, and obesity indices. This study investigates the associations between device-measured sitting, standing and different modes of physical activity (i.e., slow walking, brisk-walking, cycling and high-intensity activity) and measured body mass index (BMI) in children using person-based latent profile analyses and Partial Least Squared-structural equation modeling (PLS-SEM).

**Methods:**

A total of 344 children (11.5 ± 0.81 years, boys *n* = 139) wore a triaxial accelerometer (Fibion®) on their thigh for eight days, and their weight and height were measured at school. Latent profile analysis formed profiles including BMI, total sitting time, and physical activities, and their associations were further studied with PLS-SEM.

**Results:**

The latent profile analysis indicates that high levels of physical activity always coincide with low sitting time. Both normal weight and overweight/obesity can coexist with low physical activity and prolonged sitting. The PLS-SEM results highlight a cascade-like sequence in the relationship between various types of physical activity, sitting time, and BMI. This sequence begins with light-intensity activities, such as standing, progresses to higher-intensity activities, and ultimately through reduced sitting time (sample mean= -0.01; effect size = 0.0001; *p* = 0.02), mediates a decline in BMI (sample mean= -0.06; effect size = 0.0036; *p* = 0.01). The most positive effects on sitting time and BMI occur when this pattern is adhered to consistently, suggesting that omitting steps could negatively impact the associations.

**Conclusion:**

These findings suggest that persuading children to increase physical activity incrementally, starting from low-intensity activities such as standing and slow walking to activity types with higher intensities, possibly influence BMI by mediating reduced sitting time. This approach is particularly inclusive for overweight and obese children, taking into account the potential challenges they may encounter when performing activity types with high intensity. These cross-sectional associations need to be verified with longitudinal and experimental designs.

**Supplementary Information:**

The online version contains supplementary material available at 10.1186/s44167-024-00053-9.

## Introduction

The associations between device-based measurements of physical activity, sitting time and obesity indices in children and adolescents are reported to be mixed and potentially complex [1–3]. Most studies in this area have primarily focused on moderate to vigorous physical activity (MVPA). However, simply focusing on MVPA has been criticized as it represents only a small fraction of daily physical activity in children and adolescents, about 20 to 25 min, or 4–5% [4]. As a result, it has been suggested that this small proportion of MVPA cannot fully explain the relationship between physical activity, sedentary time, and adiposity [1, 2, 4–8]. Instead, non-exercise daily activities such as light-intensity physical activities (e.g., standing, walking) and sitting or lying time are considered the primary determinants of total daily energy expenditure [9–11]. Therefore, the comprehensive spectrum of physical activity (i.e., sitting, light and moderate to vigorous intensities) should be included in analyses to accurately classify individuals and reveal the relationship between the constituents of total physical activity and adiposity in young people [1, 5–7, 12].

Past studies have typically examined the relationship between physical activity at different intensity levels (e.g., moderate-to-vigorous activity) and obesity without taking into account the specific physical activities (such as standing, walking, or cycling) [13, 14]. This approach could potentially be problematic since obese children engage in weight-bearing physical activities at relatively higher intensity due to their higher body mass [15], which might lead to underestimated physical activity levels in these children. Additionally, discussing physical activities at different intensities is more straightforward. For instance, “walking or cycling for half an hour” is easier to comprehend than “doing moderate-to-vigorous activity” [16]. Thus, understanding the role of physical activities in health can produce results that are easier to communicate and interpret for the general public [16].

Many studies have inaccurately classified some light-intensity activities, such as standing and slow walking, as sedentary because they have defined sedentary time as a lack of ambulation (i.e., a low number of impacts or counts) rather than considering the postural element [1, 3, 17]. Triaxial thigh-worn accelerometry has been suggested to more accurately capture posture (e.g., standing, sitting), activity type (e.g., cycling, running, walking), and activity intensity compared to wrist and hip placements over a 24-hour cycle [18–21]. However, only a few studies have utilized thigh-worn accelerometry to explore the associations between sitting time, physical activities at different intensities, and adiposity in young people [5].

Advanced statistical methods might reveal more specific associations between sitting, physical activities, and obesity compared to traditional statistical methods [5, 19, 22–24]. Traditional statistical methods, such as regression models and analysis of variance, can only elucidate associations between variables or the effects of one variable on another without accounting for individual differences. This does not allow for more complex associations between body mass index (BMI), sitting time, and physical activity. However, a person-centered approach, such as latent profile analysis, groups individuals based on their similar characteristics and can provide more insight, particularly in populations with diverse characteristics such as children or adolescents [22–24]. Furthermore, partial least squares-structural equation modeling (PLS-SEM) is an intriguing method for investigating complex physical activity typologies related to different health measures. It examines a sequence of multiple dependent and independent variable blocks, creating more intricate paths of associations among variables [19, 25].

The objective of this study is to examine associations between device-measured sitting, standing and different modes of physical activity (i.e., slow walking, brisk-walking, cycling and high-intensity activity), and BMI in a sample of 10-12-year-old children using person-centered statistical methods (i.e., latent profile analysis) and PLS-SEM.

## Methods

The current cross-sectional study was conducted according to the Declaration of Helsinki and the study protocol received approval from the Aalto University Research Ethics Committee on October 10, 2019, and data collection was undertaken in Spring 2021 during the snow-free period. Data were gathered from 10-13-year-old children residing in two South-Eastern Finnish cities, Mikkeli and Kouvola, as part of the Freeride-project [26, 27]. Children and their parents were approached through 11 primary schools in Mikkeli and 10 in Kouvola, engaging a total of 331 children in Mikkeli and 369 in Kouvola. With the Department of Education approval for the study, we were authorized to contact school principals directly. Principals, in turn, provided permission to approach teachers. Through the teachers, study information sheets and informed consent forms were distributed and sent home with the children to be signed by their parents or guardians. Out of these, 361 children returned informed consent forms signed by their parents and demonstrated willingness to participate based on their oral consent. However, due to withdrawals or incomplete data (*n* = 5), removal of missing values (*n* = 8), or outliers (*n* = 4), the final sample size for statistical analysis reduced to 344 participants.

### Measurements

BMI: Weight was recorded in underwear and without shoes using an electronic scale (Type SECA 861) to the nearest 0.1 kg. Height was gauged barefoot in the Frankfurt horizontal plane using a telescopic height measuring instrument (Type SECA 225) to the nearest 1 mm. BMI was then calculated as body weight in kilograms divided by the square of height in meters. The International Obesity Task Force (IOTF) BMI cut-off points were utilized to define underweight, normal weight, overweight, and obesity [28, 29].

Physical activity and sitting: For accelerometer measurement, children were requested to note their waking and sleeping times, any non-wear periods, as well as instances of atypical days (e.g., due to illness) [27]. Using this information, only waking hours of accelerometry reports were used. At school, the researcher aided the children in wearing the accelerometers, provided additional medical adhesives, and instructed on their proper use. This was performed one child at a time, either during recess or the next lesson. The Fibion® device (Fibion Inc, Jyväskylä, Finland) was worn by children for eight days, 24 h each day. The device was affixed vertically at the centerline and horizontally at the upper third level on the anterior side of the thigh, secured in a waterproof covering with medical adhesive tape. The Fibion® device measures raw acceleration on three axes with an internal sampling rate of 12.5 Hz. It does not have buttons or a display and can function for about 30 days on a full charge. Fibion data output includes time spent sitting, standingand different modes of physical activity, and their energy expenditure. For the present study, the following outcomes were used for further analysis:

1. Sitting.
2. Standing.
3. Slow walking (walking at < 3.5 METs intensity).
4. Brisk walking (walking at > 3.5 METs intensity).
5. Cycling.
6. High intensity activity (any activity at > 7 METs intensity, like running, that does not fall into the other categories).

Fibion® is validated for estimating moderate-to-vigorous physical activity and energy expenditure against indirect calorimetry [30]. Fibion® has been demonstrated to have an overall accuracy of 85-89% in detecting different physical activities, with high accuracy (94-100%) for detecting prone and supine lying, sitting, and standing [21]. Furthermore, Fibion® shows good to excellent validity for measuring sedentary (sitting) and upright (standing and walking) time against the ActivPAL4 monitor [21, 31]. Fibion also has proven capability to detect free-living cycling activity in children [20].

### Statistical analysis

Before further analysis, all data were checked for normality, skewness, kurtosis, and outliers. All accelerometer variables (i.e., sitting, standing, slow-walking, brisk-walking, cycling, and high-intensity activity) deviated from normality, so these variables were log-transformed using the “rcompanion” package [32]. The “jmv” package was employed to compare means via an analysis of variance [33].

Latent profile analysis was performed using R packages “tidyverse” and “tidyLPA” to categorize children based on sitting, standing, and different modes of physical activity (i.e., slow walking, brisk-walking, cycling, and high-intensity activity), and BMI [34, 35]. The selection of the number of classes was informed by four model fit indices: Bayesian information criterion (BIC), Akaike information criterion (AIC), the bootstrapped likelihood ratio test (BLRT), and entropy [23]. A model with lower BIC and AIC, entropy approaching 1, and a *p*-value < 0.05 indicates a better profile. For entropy, a value of > 0.80 indicates highly discriminating latent classes. For BLRT, a *p*-value < 0.05 implies that a k class model is superior to a later class model [23].

To examine the direct and indirect associations among BMI, physical activities at different intensities, and sitting, after controlling for sex, we applied PLS-SEM. Prior to performing PLS-SEM, a confirmatory factor analysis (CFA) was conducted between latent variables. The goodness of fit was assessed using a two-index presentation strategy [36]. In this strategy, the Standardized Root Mean Square Residual (SRMR) should be < 0.08 and is paired with at least one or more other absolute indexes to indicate goodness of fit [36, 37]. Thus, we used the Normed Fit Index (NFI), where a value closer to 1 indicates a better fit [38]. Construct reliability and validity (CRV) were evaluated according to Henseler et al.‘s (2009) suggestions, where Cronbach’s Alpha should be > 0.7 and Average Variance Extracted (AVE) should be > 0.5, and composite reliability should range from 0.7 to 0.95 to indicate good convergent validity [37, 39]. For interpreting indirect effects, we utilized the effect size (here V effect size) for indirect associations, calculated by the square of the sample mean, with 0.01, 0.075, and 0.35 indicating small, medium, and large effect sizes, respectively [40].

Except for PLS-SEM, which was conducted using the commercial software SmartPLS, the rest of the statistical analyses were performed using RStudio Version R-4.1.2 for Windows. Statistical significance was set at *p* < 0.05 (two-tailed).

## Results

### General characteristics of participants

The mean total waking wear time of Fibion data (i.e., sitting, standing, and different modes of physical activity) was 15.96 ± 1.57 h/day. Boys had higher brisk-walking (*p* < 0.01) and high-intensity activity duration (*p* < 0.01) compared to girls. Conversely, girls had higher slow-walking (*p* = 0.01) and standing duration (*p* < 0.01) compared to boys. No significant differences were observed in BMI, sitting time, and cycling time by sex.

### Latent profile analysis

**Fig. 1:**
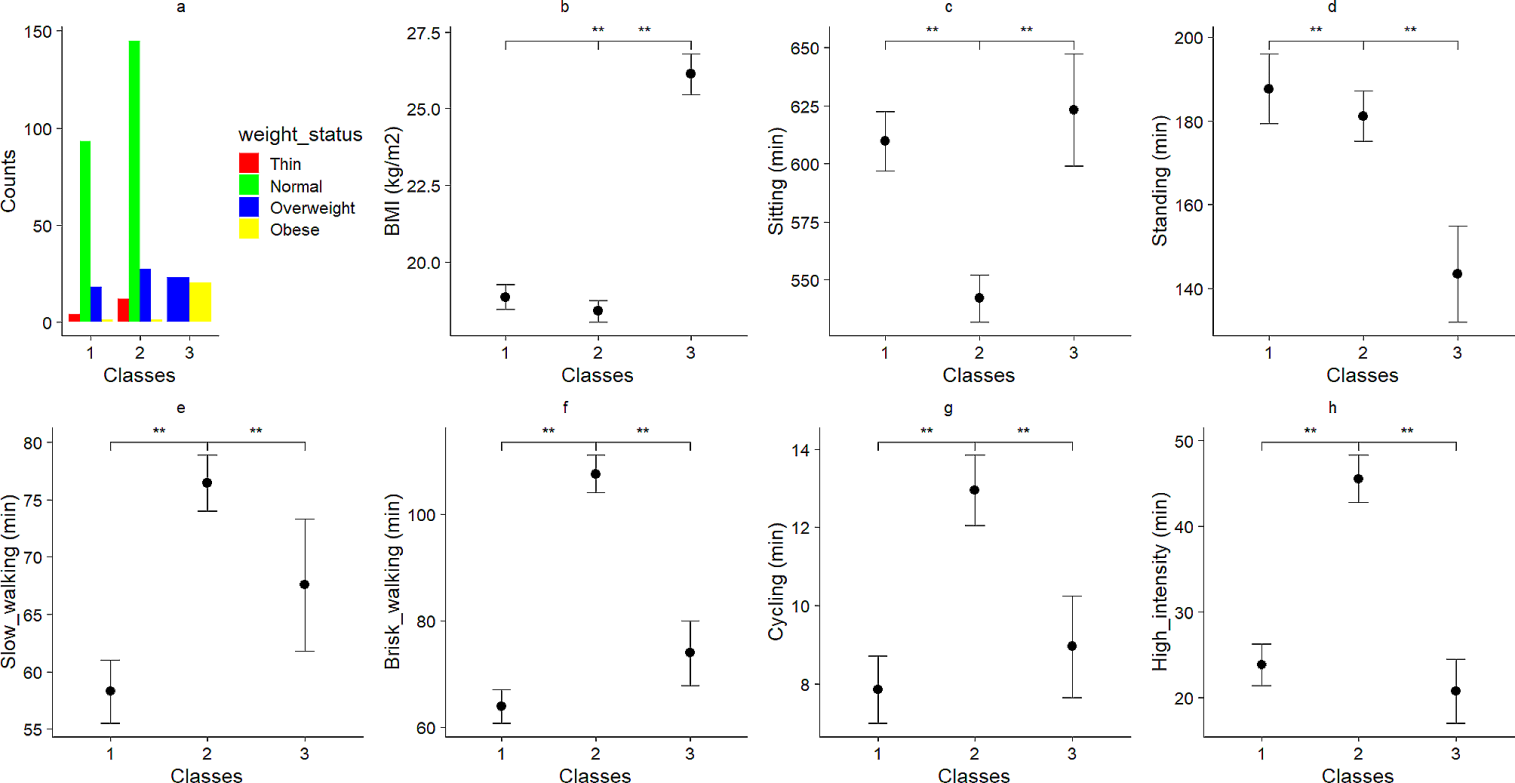
Characteristics of each class (Mean ± CI_95%_ for all figures except for “a” which shows prevalence of weight status in each class)

Profiles 1 through 4 were compared to identify the optimal number [23]. The 3-class profile was selected, exhibiting the lowest BIC = 6666.71 and highest Entropy = 0.78 [23]. Standardized group averages on various physical activities at different intensities for the three-profile solution are depicted in Supp Fig. 1. The proportion of children in each profile and the prevalence of weight status categories are shown in Fig. 1.

### Categorizing children according to different classes

Latent profile analysis results (Fig. 1 and Supp Fig. 1) categorized children as follows:

Class 1 = Normal BMI – high sitting – high standing – low slow-walking – low brisk-walking – low cycling – low high-intensity (i.e., normal weight – high sitting – low active).

Class 2 = Normal BMI – low sitting – high standing – high slow-walking – high brisk-walking – high cycling – high high-intensity (i.e., normal weight – low sitting – high active).

Class 3 = High BMI – high sitting – low standing – low slow-walking – low brisk-walking – low cycling – low high-intensity (i.e., overweight/obese – high sitting – low active).

The prevalence of different weight status groups among children is shown in Fig. 1a.

### Partial least squares-structural equation modeling (SEM)

To reduce the number of variables, we first performed a factor analysis. Latent variables included in the confirmatory factor analysis were derived from previous studies and were a combination of standing and slow-walking time (i.e., light intensity activities) as one latent variable, and a combination of brisk-walking, cycling, and high-intensity time as another latent variable (i.e., moderate-to-vigorous intensity activities) [1, 5–7, 12]. Results of CFA indicated a poor fit model for both latent variables (SRMR = 0.14, Chi-Square = 18.13, NFI = 0.41). We then checked the CRV [37, 39]. For both brisk-walking + cycling + high-intensity (Cronbach’s Alpha = 0.55, Composite reliability = 0.69, AVE = 0.50) and standing + slow-walking (Cronbach’s Alpha = 0.29, Composite reliability = 0.61, AVE = 0.52), the CRV indicated poor reliability and validity. In the next step, we sought to determine which variable should be removed from each construct. Bootstrapping results indicated no significant values for standing in the latent standing + slow-walking (T statistics = 0.36, *p* = 0.72) or high-intensity in brisk-walking + cycling + high-intensity construct (T statistics = 1.41, *p* = 0.16). Consequently, we divided standing + slow-walking into two observed variables (i.e., standing and slow-walking), and brisk-walking + cycling + high-intensity into two variables, one observed (i.e., high-intensity) and one latent variable including brisk-walking + cycling, which we named Brisk-walking/Cycling.

The first construct of the model showed an acceptable fit (SRMR = 0.07, Chi-Square = 85.78, NFI = 0.85; Supplementary Fig. 2). We then proceeded to remove the nonsignificant paths in the model. After doing so, the model fit remained acceptable (SRMR = 0.07, Chi-Square = 92.87, NFI = 0.84). No collinearity effect was observed for the variables in the final model (i.e., inner and outer VIFs were between 1.00 and 1.46).

#### Direct associations

**Fig. 2:**
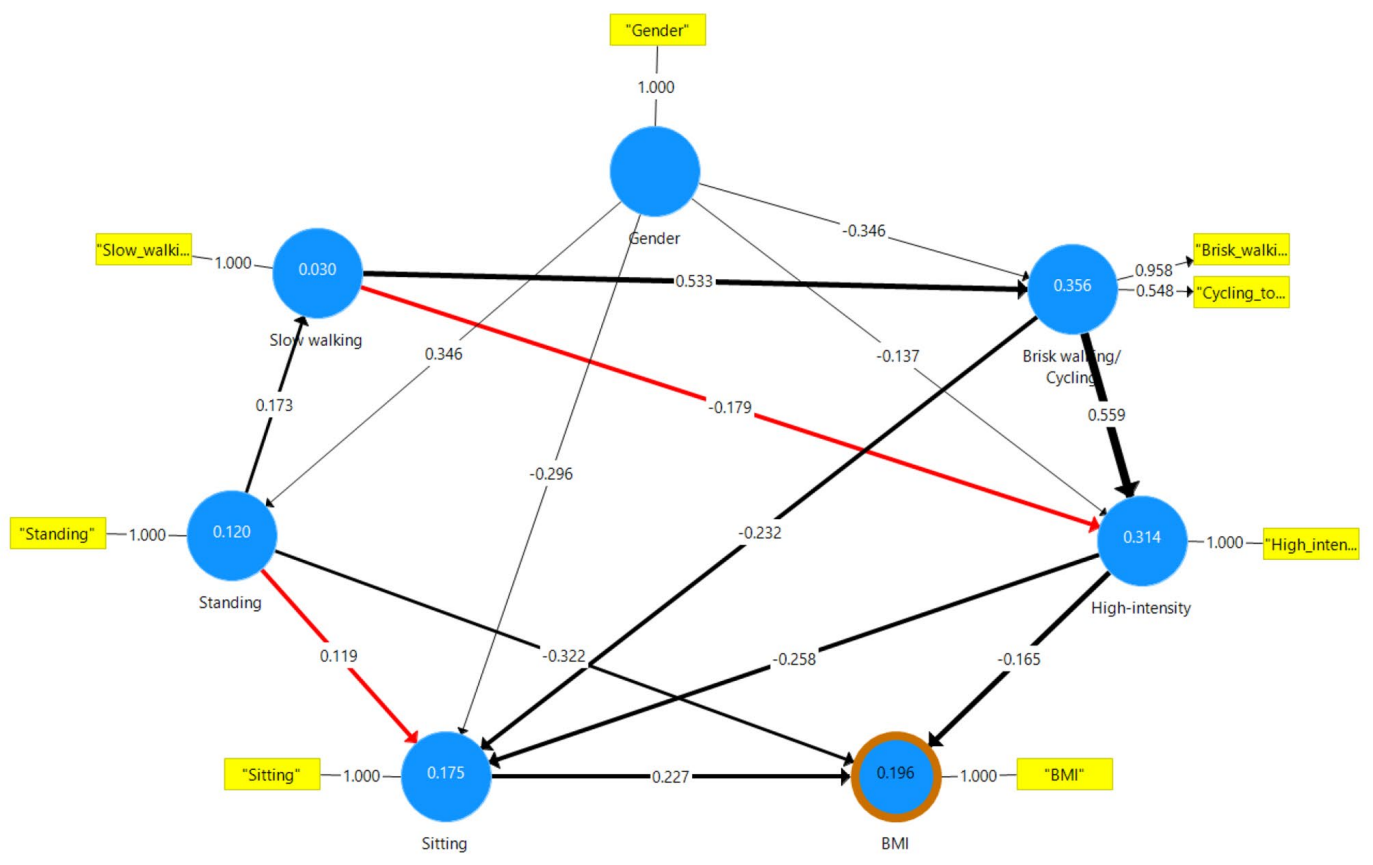
PLS-SEM predicting BMI in children after removing non-significant associations

A negative association was found between high-intensity activity time and BMI (β= -0.17, T statistics = 2.82, f Square = 0.03, *p* < 0.01; Fig. 2), but this association was mediated by sitting time (sample mean= -0.06; effect size = 0.0036; *p* = 0.01; Supplementary Table 2). A positive association was revealed between sitting time and BMI (β = 0.23, T statistics = 4.44, f Square = 0.06, *p* < 0.01). Standing was negatively associated with BMI (β= -0.32, T statistics = 7.08, f Square = 0.13; *p* < 0.01), but positively associated with sitting (β = 0.12, T statistics = 2.10, f Square = 0.01, *p* < 0.01), and the association between standing and BMI was mediated by sitting time (sample mean = 0.03; effect size = 0.0009; *p* = 0.05).

A negative association was revealed between high-intensity activity time (β= -0.26, T statistics = 3.83, f Square = 0.06, *p* < 0.01), and brisk walking/cycling (β= -0.23, T statistics = 3.74, f Square = 0.04, *p* < 0.01) with sitting time. Finally, a direct association was found between brisk walking/cycling with high-intensity (β= -0.56, T statistics = 10.36, f Square = 0.29, *p* < 0.01) and slow-walking (β= -0.53, T statistics = 12.43, f Square = 0.43, *p* < 0.01).

In summary, the more intense the physical activity types, the more directly they were associated with sitting time or BMI. However, all physical activity types, either with light or high intensity, were associated with each other, and this association strengthens as the intensity of activity types increased step by step from lower to higher intensity. For example, there was no association between standing and either brisk walking or high-intensity, only between standing and slow-walking. Moreover, slow walking was positively associated with brisk walking but negatively associated with high intensity. The same relationship also existed for standing when standing alone was directly and positively associated with sitting time but indirectly and negatively associated with sitting time when it was in cascade of a step-by-step increments of physical activity types to higher intensities (supplementary Tables 1 and 2).

#### Indirect associations

We observed a weak negative indirect association between brisk walking/cycling time and BMI (sample mean= -0.178; effect size = 0.03; *p* < 0.01), mediated by sitting time (sample mean= -0.05; effect size = 0.0025; *p* = 0.01). Brisk walking also showed a negative indirect association with sitting time, mediated by high-intensity physical activity time (sample mean= -0.14; effect size = 0.0196; *p* < 0.01). Slow-walking time was indirectly and negatively associated with BMI (sample mean= -0.054; effect size = 0.003; *p* < 0.01) only through a step-by-step physical activity types cascade (slow walking -> brisk-walking/cycling) model. However, when removing brisk-walking/cycling, the association reversed (sample mean = 0.01; effect size = 0.0001; *p* = 0.05; supplementary Table 2). Brisk walking/cycling time and sitting time were indirectly and negatively associated with each other (sample mean= -0.144; effect size = 0.020; *p* < 0.01). Similarly, a weak negative indirect association was found between slow-walking time (sample mean= -0.154; effect size = 0.024; *p* < 0.01) or standing time (sample mean= -0.027; effect size = 0.0007; *p* < 0.01) and sitting time. However, this association reversed when removing the step-by-step cascade (i.e., removing brisk walking/cycling).

Finally, a weak positive indirect association was found between standing time and brisk walking/cycling time (sample mean = 0.092; effect size = 0.008; *p* < 0.01). When removing the cascade increments of physical activity types for higher intensities (i.e., removing slow walking), this association reversed. A medium positive indirect association was revealed between slow-walking time and high-intensity activity time (sample mean= -0.297; effect size = 0.088; *p* < 0.01).

In summary, all physical activity types at different intensities are indirectly associated with BMI or sitting time. It appears that this association follows a step-by-step cascade-like pattern, and removing one step negatively affects the model aimed at decreasing sitting time and BMI. The interconnectedness of these activity types underscores the importance of considering all physical activity types with different intensity levels in the model.

## Discussion

In this study, we examined the associations between BMI, sitting, standing and different modes of physical activity (i.e., slow walking, brisk-walking, cycling and high-intensity activity) in a sample of 10–12-year-old children. The results of latent profile analysis suggest that high sitting time invariably coexists with low physical activity time at any intensity. This implies that engaging in high levels of physical activity may potentially serve as a protective factor against the adverse effects of prolonged sitting. However, both normal weight and overweight/obesity can coincide with low physical activity and high sitting time.

The PLS-SEM results revealed associations between different physical activities, sitting time, and BMI following a step-by-step, cascade-like pattern. This pattern begins with an activity type with very light intensity (i.e., standing) and escalates step by step to activities with higher intensities, with connections strengthening as activities progress from lighter to higher intensities. This cascade-like pattern impacts sitting, and decreased sitting time mediates lower BMI. However, this model is effective only when the step-by-step pattern is adhered to, meaning that skipping one step and jumping to a higher intensity activity type negatively affects the model.

To our knowledge, there are no similar studies exploring the association between physical activities at different intensities (i.e., standing, walking, cycling, and high-intensity activity) using thigh-worn triaxial accelerometers with BMI in children. We opted for physical activities as they are simpler to communicate and follow than measures such as moderate-to-vigorous intensity physical activity. However, a few studies have investigated this association using different physical activity intensities in children and adolescents [2, 5, 13, 14].

Recently, Parker et al. (2019) employed self-reported surveys to measure various activities among adolescents, including active travel to school, leisure-time sedentary behavior, sport participation, and demographic variables. Through the application of Latent Class Analysis (LCA), they discerned three distinct typologies: (1) ‘physically inactive, highly sedentary’, (2) ‘highly active and low sedentary’, and (3) ‘moderately active with high screen time’. These findings align closely with the outcomes of our own study [41]. Their research cohorts collectively demonstrated an escalation in sedentary behavior and a decline in physical activity levels with advancing age, from childhood to adolescence. The authors recommended targeted interventions aimed at reducing sedentary time and promoting increased physical activity [14, 41].

The correlation between physical activity, sedentary time, and adiposity in children and adolescents is considered complex [1]. Biddle et al. (2018) reported a weak association between TV viewing and adiposity in children and adolescents but found inconsistent associations between device-based measures (i.e., accelerometry) of sedentary time and adiposity. A meta-analysis reported a small but significant decrease in BMI when interventions reduced sedentary behavior, particularly in overweight and obese children and adolescents [42]. Some other authors reported a mixed results when considering the relationship between intensity of physical activity and adiposity. Certain studies have cited MVPA as an independent predictor of adiposity in children and adolescents [43, 44], while others have underscored the protective role of light-intensity activities against high adiposity [1, 5]. Biddle et al. 2018 argued that adiposity and sedentary behaviors might be correlated, but this relationship can be confounded by factors such as MVPA, standing, slow walking, sleep, and dietary patterns.

In our study, the latent profile analysis showed that not only normal weight but also overweight children can be either highly active or low active, with high or low sitting time. However, obesity was exclusively associated with high sitting time and low activity time. Biddle et al. recently also demonstrated that the relationship between adiposity and sitting time depends on time spent in total-light intensity physical activity and light-light intensity activities such as standing and slow walking, but not on MVPA or high-light-intensity physical activity in a sample of adolescents [5]. They emphasized the importance of light-intensity physical activity in mitigating the negative effects of prolonged sitting on adiposity in adolescents [5]. Accordingly, several authors have highlighted the necessity of light-intensity physical activity and proposed an integrative model that includes different types and intensities of physical activity, not just MVPA [5–7].

Results of the latent profile analysis indicated that overweight or obese children (class 3) had low standing time and high sitting time. However, class 1, with a high rate of normal weight children, had high sitting time but also high standing time. Furthermore, the PLS-SEM results revealed a direct negative association between standing time and BMI. These findings suggest that overweight and obese children stand less than their normal weight peers. This might occur due to their higher body mass and adiposity, which deter them from standing for extended periods in daily life [1]. This supports the bidirectional association between adiposity and physical activity, in which high adiposity possibly inhibits activity [15]. Moreover, the results of PLS-SEM indicated a direct negative association between standing time and BMI. Still, this association is negatively mediated with higher sitting, suggesting that only high standing can increase sitting time and lead to an increased BMI. Conversely, we observed a negative indirect association (i.e., through physical activities at higher intensities) between standing and BMI. These results suggest that light-intensity physical activity types, such as standing time, are negatively associated with sitting time only when they are part of other more intensive physical activities. However, given the cross-sectional nature of this study, longitudinal studies are required to examine whether standing alone or combined with other physical activity types can alter sitting time or adiposity in children.

Given the likely complex interplay between sedentary time and physical activities, it is crucial to consider the types and intensities of activities that replace sedentary time in interventions [1, 2]. Notably, during waking hours, any reduction in sitting time will primarily result in an increase in light-intensity physical activity types such as standing and slow walking [1], but may also include MVPA [45]. It’s been demonstrated that even movements such as increased standing can enhance energy expenditure [46, 47] and positively impact cardiometabolic biomarkers [48].

Some studies have reported the coexistence of high sitting time and high-intensity physical activity among youth [49]. For instance, using cluster analysis, Marshall et al. suggested a coexistence of high sitting time and MVPA [49]. However, the latent profile analysis employed in our study did not confirm the coexistence of high sitting time and/or high BMI and high levels of any physical activity types at different intensities in children, which is according to a recent similar study by Parker et al. (2022) where they used similar methods [41]. Moreover, in our study, we noted that the class with the highest prevalence of overweight and obesity not only had high sitting time but also low standing time, walking, cycling, and high-intensity physical activity time. This finding aligns with the literature showing the connection between obesity, high sedentary time, and low levels of physical activity [5–7, 50–52].

Finally, the results of latent profile analysis revealed a coexistence of low levels of all physical activities at different intensities and high sitting time in children with either high BMI or normal BMI. This suggests that not only overweight/obese children but also a group of normal weight children can exhibit high sitting time accompanied with low physical activity time. Therefore, programs aimed at increasing physical activity time should consider all children, not just those who are overweight and obese [53].

Although we found a direct negative association between high-intensity activity time and BMI, this relationship was mediated by a sequential cascade of activity types from lower to higher intensities and was ultimately moderated by reduced sitting time. Research has shown that focusing solely on high volumes of high-intensity physical activity can lead to unpleasant experiences and feelings [54]. Moreover, prescribing only high-intensity physical activity may result in a compensatory decrease in lower-intensity activities such as standing and slow walking and an increase in sitting time [55]. It may be more practical to reduce sitting time by increasing activities such as standing and walking, which represent the most variable components of daily total energy expenditure [56], to reduce adiposity [2, 57]. Additionally, beginning with low-intensity activities could be more motivational, easier, and more practical than starting from MVPA for preventing obesity and sedentariness in children and adolescents, especially those who are overweight or obese [5, 58–60]. For instance, starting to target sedentary behavior [61] through active pedagogy or height-adjustable desks (i.e., standing lessons) and active breaks, in the school classroom could be practical methods for integrating light-intensity physical activities, thereby increasing energy expenditure while reducing sitting time and adiposity in children and adolescents [47, 61–64].

Our study results, based on PLS-SEM analysis, revealed that the intensity of physical activities directly corresponds with their association with sitting time or BMI. However, we discovered that all physical activities, regardless of their intensity, exhibit either direct or indirect associations with each other, and these connections intensify as activities transition from lighter to higher intensities. For instance, standing showed no connection with brisk walking or high-intensity activities, but it was linked to slow walking. A direct positive association was observed between slow walking and brisk walking, while a negative association was evident with high-intensity activities. These findings emphasize the significance of physical activities of various intensities and their interconnectedness, which ultimately influences sitting time and BMI.

Recently, Dunstan et al. (2021) suggested a “staircase approach,” especially for healthy individuals younger than 45 years. This approach advocates for an initial reduction in sitting time, followed by an increase in standing and slow walking, eventually leading to an increase in light-intensity activities and finally, a rise in MVPA [65]. Our findings align with this approach, providing empirical evidence that light-intensity activities seem to serve as a necessary precursor to higher intensities, mediating BMI by reducing sitting time. Removing light-intensity activities could potentially disrupt this model, thereby reducing the effectiveness of interventions aimed at reducing sitting time and BMI.

**Fig. 3:**
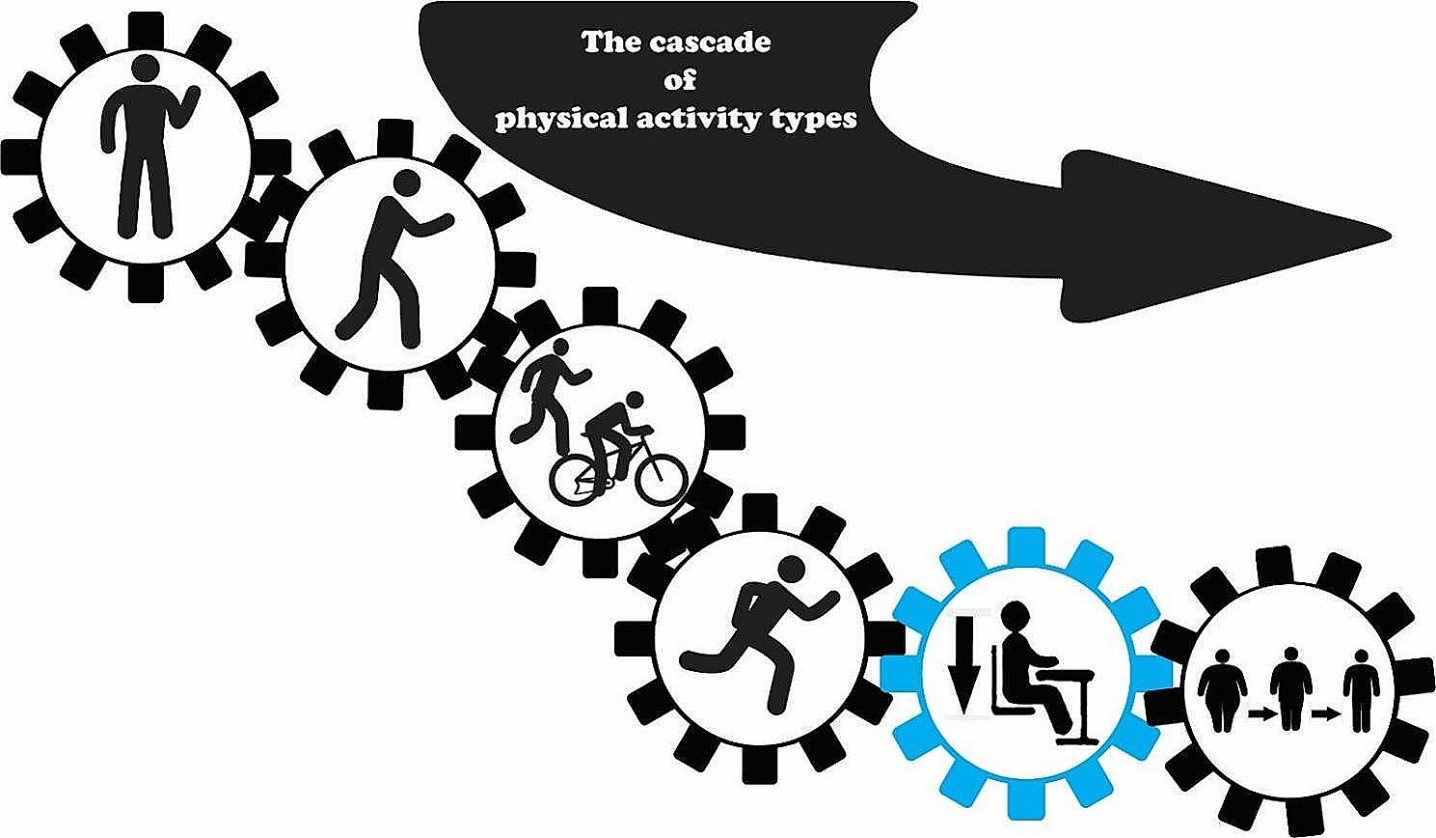
This illustration synthesizes findings from Fig. 2, detailing the cascade of physical activity types in children. The results suggest that physical activity regimen possibly be effective if typically commences with light-intensity activities, such as standing, gradually advancing towards actions like slow-walking. The gradual progression appears to trigger a cascading effect, facilitating a smoother transition to higher-intensity activities. For example, a child who has previously engaged in a blend of standing, slow-walking, and brisk walking activities will find it simpler to start running than his peer with no physical activity regimen. Bypassing any step in this sequence can disrupt this progression. Notably, while no single activity type has a direct link to BMI, this gradual approach ultimately results in reduced sitting time, thereby mediating a decrease in BMI. The results of the latent profile analysis substantiate this concept, indicating that an elevated overall physical activity duration serves as a protective factor against prolonged sitting time.

The PLS-SEM results suggest a mechanistic interplay, where engaging in light-intensity activities may set the stage for higher intensity exercises, akin to the interconnected gears in a machine, each one propelling the next to keep the whole system in motion. (Fig. 3). As intensity levels increase, the relationships between activity types, sitting time, and BMI become stronger. Each intensity level appears to be crucial in maintaining the desired effects on reducing sitting time and BMI. If a step in this cascade, such as brisk walking/cycling, is removed, the associations reverse (i.e., results of supplementary Table 3). This is according to recent study results, where the authors showed that total light intensity physical activity and low light intensity physical activity but not high-light intensity physical activity or MVPA possibly protect deleterious effects of sitting on adiposity in adolescents [5]. This underscores the importance of including a full spectrum of physical activity types to effectively reduce sitting time and BMI (Fig. 3). Overall, the study underscores the value of a balanced, multi-intensity approach to physical activity for positive adiposity outcomes among children. However, longitudinal and experimental studies are needed to validate this model and determine its success in decreasing sitting time and adiposity in children and adolescents.

### Strengths and limitations

The strengths of our study include the use of device-based measures of sitting and physical activities at different intensities and sitting [31, 66], along with the application of person-centered and PLS-SEM analysis methods [23, 24] which have been recommended for studies exploring the association between physical activity, adiposity, and sitting time [5–7, 19]. However, the cross-sectional nature of the study, the use of BMI instead of direct measures of adiposity, and the lack of socioeconomic status information, sleep and dietary patterns data limit our findings. Moreover, due to the exploratory nature of the analyses, the sample size was not predetermined for the current analyses. Therefore, these findings should be interpreted with caution. They are not deterministic but rather serve to generate new insights and hypotheses. These hypotheses should be tested in future studies with appropriately powered, preferably longitudinal and experimental, designs.

## Conclusion

In summary, our findings suggest that high sitting time consistently coincides with low physical activity time across all intensities and types, whereas greater physical activity time always coincides with low sitting time. However, both healthy weight and overweight/obese conditions can coexist with low physical activity levels and high sitting time. We observed no coexistence of high sitting time and/or high BMI with high levels of any physical activities at different intensities. Based on our cross-sectional analysis, we identified a clear cascade-like pattern associating various physical activities at different intensities with sitting time and BMI. The data suggests that initiating with light-intensity activities, such as standing, can serve as a pivotal foundation for transitioning to more strenuous exercises. This structured progression appears to offer potential benefits in terms of reducing both sitting time and BMI. Furthermore, this step-by-step approach considers the challenges overweight and obese children may face when performing MVPA and recognizes the potential difficulty and unfamiliarity children may have with such activities.

## Electronic supplementary material

Below is the link to the electronic supplementary material.

Supplementary Material 1

## Data Availability

The datasets used and/or analysed during the current study are available from the corresponding author on reasonable request.

## Abbreviations

BMI: Body mass index
CFA: confirmatory factor analysis
MVPA: Moderate to vigorous physical activity
PLS-SEM: Partial Least Squared-structural equation modeling
SRMR: Standardized Root Mean Square Residual
NFI: Normed Fit Index
CRV: Construct reliability and validity
AVE: Average Variance Extracted

## References

[1] Biddle et al. Sedentary behaviors and adiposity in young people: causality and conceptual model. Exerc Sport Sci Rev. 2018;46. 10.1249/JES.0000000000000135 28885266

[2] Bourdier P et al. The role of physical activity in the regulation of body weight: the overlooked contribution of light physical activity and sedentary behaviors. Obes Rev. 2022;e13528. 10.1111/obr.13528 PMC10910694 36394185

[3] Cliff DP, Hesketh KD, Vella SA, Hinkley T, Tsiros MD, Ridgers ND et al. Objectively measured sedentary behaviour and health and development in children and adolescents: systematic review and meta-analysis. Obes Rev. 2016;17. 10.1111/obr.12371 26914664

[4] Australian Bureau of Statistics. Australian Health Survey: Physical Activity. Aust. Bur. Stat. 2013.

[5] Contardo Ayala AM, Salmon J, Dunstan DW, Arundell L, Timperio A. Does light-intensity physical activity moderate the relationship between sitting time and adiposity markers in adolescents? J Sport Heal Sci. 2022;11. 10.1016/j.jshs.2020.04.002 PMC9532587 32407803

[6] Al D. et. Light-intensity physical activity is associated with adiposity in adolescent females. Med Sci Sports Exerc. 2014;46. 10.1249/MSS.0000000000000357 24797308

[7] Dowd K, Harrington D, Hannigan A, Purtill H, Kelly SM, Macken AP et al. The development of activity profiles in adolescent females and their association with adiposity. Pediatr Exerc Sci. 2016;28. 10.1123/pes.2015-0081 26252370

[8] Troiano RP, Berrigan D, Dodd KW, Mâsse LC, Tilert T, Mcdowell M. Physical activity in the United States measured by accelerometer. Med Sci Sports Exerc. 2008;40. 10.1249/mss.0b013e31815a51b3 18091006

[9] Levine JA, Vander Weg MW, Hill JO, Klesges RC. Non-exercise activity thermogenesis: the crouching tiger hidden dragon of societal weight gain. Arterioscler Thromb Vasc Biol. 2006. 10.1161/01.ATV.0000205848.83210.73 16439708

[10] Lanningham-Foster L, Levine JA. Energy expenditure in children: the role of NEAT (non-exercise activity thermogenesis). Contemp Endocrinol. 2018.

[11] Colley RC, Garriguet D, Janssen I, Craig CL, Clarke J, Tremblay MS. Physical activity of Canadian children and youth: accelerometer results from the 2007 to 2009 Canadian health measures survey. Heal Rep. 2011;22. 21510586

[12] Aadland E, Kvalheim OM, Anderssen SA, Resaland GK, Andersen LB. The multivariate physical activity signature associated with metabolic health in children. Int J Behav Nutr Phys Act. 2018;15. 10.1186/s12966-018-0707-z PMC6094580 30111365

[13] Parker KE, Salmon J, Costigan SA, Villanueva K, Brown HL, Timperio A. Activity-related behavior typologies in youth: a systematic review. Int J Behav Nutr Phys Act. 2019. 10.1186/s12966-019-0804-7 PMC6524235 31097036

[14] Parker K, Timperio A, Salmon J, Villanueva K, Brown H, Esteban-Cornejo I et al. Activity-related typologies and longitudinal change in physical activity and sedentary time in children and adolescents: the UP&DOWN Study. J Sport Heal Sci. 2021;10. 10.1016/j.jshs.2020.02.004 PMC8343008 33836977

[15] Tanaka C, Janssen X, Pearce M, Parkinson K, Basterfield L, Adamson A et al. Bidirectional associations between adiposity, sedentary behavior, and physical activity: a longitudinal study in children. J Phys Act Heal. 2018;15. 10.1123/jpah.2018-0011 30404530

[16] Allahbakhshi H, Hinrichs T, Huang H, Weibel R. The key factors in physical activity type detection using real-life data: a systematic review. Front Physiol. 2019. 10.3389/fphys.2019.00075 PMC6379834 30809152

[17] Hart TL, Ainsworth BE, Tudor-Locke C. Objective and subjective measures of sedentary behavior and physical activity. Med Sci Sports Exerc. 2011;43. 10.1249/MSS.0b013e3181ef5a93 20631642

[18] Stevens ML, Gupta N, Inan Eroglu E, Crowley PJ, Eroglu B, Bauman A et al. Thigh-worn accelerometry for measuring movement and posture across the 24-hour cycle: a scoping review and expert statement. BMJ Open Sport Exerc Med. 2020. 10.1136/bmjsem-2020-000874 PMC7768971 33408875

[19] Arvidsson D, Fridolfsson J, Börjesson M. Measurement of physical activity in clinical practice using accelerometers. J Intern Med. 2019. 10.1111/joim.12908 30993807

[20] Pesola et al. Sensitivity and specificity of measuring children’s free-living cycling with a thigh-worn Fibion® accelerometer. Front Sport Act Living. 2023;5. 10.3389/fspor.2023.1113687 PMC10242071 37287711

[21] Montoye et al. Evaluation of two thigh-worn accelerometer brands in laboratory and free-living settings. J Meas Phys Behav. 2022.

[22] Hensel DJ. Using latent profile analysis and related approaches in adolescent health research. J Adolesc Heal. 2020. 10.1016/j.jadohealth.2020.05.005 32739021

[23] Spurk D, Hirschi A, Wang M, Valero D, Kauffeld S. Latent profile analysis: a review and how to guide of its application within vocational behavior research. J Vocat Behav. 2020.

[24] Howard MC, Hoffman ME. Variable-centered, person-centered, and person-specific approaches: where theory meets the method. Organ Res Methods. 2018;21.

[25] Beran TN, Violato C. Structural equation modeling in medical research: a primer. BMC Res Notes. 2010;3. 10.1186/1756-0500-3-267 PMC2987867 20969789

[26] Pesola AJ, Hakala P, Berg P, Ramezani S, Villanueva K, Rinne T. The effects of free-fare public transportation on the total active travel in children: a cross-sectional comparison between two finnish towns. J Transp Heal. 2022;27.

[27] Pesola AJ, Hakala P, Berg P, Ramezani S, Villanueva K, Tuuva-Hongisto S et al. Does free public transit increase physical activity and independent mobility in children? Study protocol for comparing children’s activity between two Finnish towns with and without free public transit. BMC Public Health. 2020;20. 10.1186/s12889-020-8385-6 PMC7077102 32183761

[28] Cole TJ, Flegal KM, Nicholls D, Jackson AA. Body mass index cut offs to define thinness in children and adolescents: international survey. Br Med J. 2007;335. 10.1136/bmj.39238.399444.55 PMC1934447 17591624

[29] Cole TJ, Bellizzi MC, Flegal KM, Dietz WH. Establishing a standard definition for child overweight and obesity worldwide: International survey. Br Med J. 2000;320. 10.1136/bmj.320.7244.1240 PMC27365 10797032

[30] Zheng F, Yan L, Yang Z, Zhong B, Xie W. HbA1c, diabetes and cognitive decline: the english longitudinal study of ageing. Diabetologia. 2018;61. 10.1007/s00125-017-4541-7 PMC6448974 29368156

[31] Alkalih, et al. A new accelerometer (Fibion) device provides valid sedentary and upright time measurements compared to the ActivPAL4 in healthy individuals. Heliyon. 2022;8:e11103. 36281387 10.1016/j.heliyon.2022.e11103 PMC9586911

[32] Mangiafico SS. rcompanion: functions to support extension education program evaluation. Buildings. 2021.

[33] Şahin M, Aybek E. Jamovi: an easy to use statistical software for the social scientists. Int J Assess Tools Educ. 2019.

[34] Rosenberg J, Beymer P, Anderson D, van Lissa C J., Schmidt J. tidyLPA: an R package to easily carry out latent profile analysis (LPA) using open-source or commercial software. J Open Source Softw. 2018;3.

[35] Wickham H, Averick M, Bryan J, Chang W, McGowan L, François R et al. Welcome to the tidyverse. J Open Source Softw. 2019;4.

[36] Hu L-T, Bentler PM, Hoyle RH. Evaluating model fit. In R. H. Hoyle, editor, Structural equation modeling: Concepts, issues, and applications. Eval Model fit. 1995;54.

[37] Henseler J, Ringle CM, Sinkovics RR. The use of partial least squares path modeling in international marketing. Adv Int Mark. 2009;20.

[38] Bentler PM, Bonett DG. Significance tests and goodness of fit in the analysis of covariance structures. Psychol Bull. 1980;88.

[39] Hair JF, Howard MC, Nitzl C. Assessing measurement model quality in PLS-SEM using confirmatory composite analysis. J Bus Res. 2020;109.

[40] Ogbeibu S, Jabbour CJC, Gaskin J, Senadjki A, Hughes M. Leveraging STARA competencies and green creativity to boost green organisational innovative evidence: a praxis for sustainable development. Bus Strateg Environ. 2021;30.

[41] Parker KE, Salmon J, Brown HL, Villanueva K, Timperio A. Typologies of adolescent activity related health behaviours. J Sci Med Sport. 2019;22. 10.1016/j.jsams.2018.08.015 PMC6703174 30190099

[42] Stierlin AS, De Lepeleere S, Cardon G, Dargent-Molina P, Hoffmann B, Murphy MH et al. A systematic review of determinants of sedentary behaviour in youth: a DEDIPAC-study. Int J Behav Nutr Phys Act. 2015. 10.1186/s12966-015-0291-4 PMC4600309 26453175

[43] Strong WB, Malina RM, Blimkie CJR, Daniels SR, Dishman RK, Gutin B et al. Evidence based physical activity for school-age youth. J Pediatr. 2005;146. 10.1016/j.jpeds.2005.01.055 15973308

[44] Scheers T, Philippaerts R, Lefevre J. Objectively-determined intensity- and domain-specific physical activity and sedentary behavior in relation to percent body fat. Clin Nutr. 2013;32. 10.1016/j.clnu.2013.03.014 23591153

[45] Verswijveren SJJM, Ridgers ND, Martín-Fernández JA, Chastin S, Cerin E, Chinapaw MJM et al. Intervention effects on children’s movement behaviour accumulation as a result of the Transform-Us! school- and home-based cluster randomised controlled trial. Int J Behav Nutr Phys Act. 2022;19. 10.1186/s12966-022-01314-z PMC9261108 35799258

[46] Mansoubi M, Pearson N, Clemes SA, Biddle SJH, Bodicoat DH, Tolfrey K et al. Energy expenditure during common sitting and standing tasks: examining the 1.5 MET definition of sedentary behaviour. BMC Public Health. 2015;15. 10.1186/s12889-015-1851-x PMC4448542 26021449

[47] Minges KE, Chao AM, Irwin ML, Owen N, Park C, Whittemore R et al. Classroom standing desks and sedentary behavior: a systematic review. Pediatrics. 2016. 10.1542/peds.2015-3087 PMC4732360 26801914

[48] Carson V, Ridgers ND, Howard BJ, Winkler EAH, Healy GN, Owen N et al. Light-intensity physical activity and cardiometabolic biomarkers in US adolescents. PLoS ONE. 2013;8. 10.1371/journal.pone.0071417 PMC3739773 23951157

[49] Marshall SJ, Biddle SJH, Sallis JF, McKenzie TL, Conway TL. Clustering of sedentary behaviors and physical activity among youth: a cross-national study. Pediatr Exerc Sci. 2002;14.

[50] Collings PJ, Wijndaele K, Corder K, Westgate K, Ridgway CL, Dunn V et al. Levels and patterns of objectively-measured physical activity volume and intensity distribution in UK adolescents: the ROOTS study. Int J Behav Nutr Phys Act. 2014;11. 10.1186/1479-5868-11-23 PMC3936923 24564949

[51] Fridolfsson J, Buck C, Hunsberger M, Baran J, Lauria F, Molnar D et al. High-intensity activity is more strongly associated with metabolic health in children compared to sedentary time: a cross-sectional study of the I.Family cohort. Int J Behav Nutr Phys Act. 2021;18. 10.1186/s12966-021-01156-1 PMC8261968 34229708

[52] Lätt E, Mäestu J, Rääsk T, Jürimäe T, Jürimäe J, Ortega FB. Vigorous physical activity rather than sedentary behaviour predicts overweight and obesity in pubertal boys: a 2-year follow-up study. Scand J Public Health. 2015;43. 10.1177/1403494815569867 25740617

[53] Adamo KB, Colley RC, Hadjiyannakis S, Goldfield GS. Physical activity and sedentary behavior in obese youth. J Pediatr. 2015;166. 10.1016/j.jpeds.2015.01.001 25684088

[54] Hoydal et al. Experiencing good results promotes positive feelings to high-intensity exercise among young adults: a qualitative study. Front Sport Act Living. 2022;4. 10.3389/fspor.2022.959079 PMC9709123 36465579

[55] Melanson EL, Keadle SK, Donnelly JE, Braun B, King NA. Resistance to exercise-induced weight loss: compensatory behavioral adaptations. Med Sci Sports Exerc. 2013;45. 10.1249/MSS.0b013e31828ba942 PMC3696411 23470300

[56] Villablanca PA, Alegria JR, Mookadam F, Holmes DR, Wright RS, Levine JA. Nonexercise activity thermogenesis in obesity management. Mayo Clin. Proc. 2015. 10.1016/j.mayocp.2015.02.001 25841254

[57] Holliday A, Burgin A, Fernandez EV, Fenton SAM, Thielecke F, Blannin AK. Points-based physical activity: a novel approach to facilitate changes in body composition in inactive women with overweight and obesity. BMC Public Health. 2018;18. 10.1186/s12889-018-5125-2 PMC5816513 29454318

[58] Biddle S. Motivation for physical activity and weight management. Int J Obes. 1998;22. 9778095

[59] Smith L, Ekelund U, Hamer M. The potential yield of Non-exercise physical activity energy expenditure in public health. Sport Med. 2015. 10.1007/s40279-015-0310-2 25648364

[60] Teixeira PJ, Carraça EV, Markland D, Silva MN, Ryan RM. Exercise, physical activity, and self-determination theory: a systematic review. Int J Behav Nutr Phys Act. 2012. 10.1186/1479-5868-9-78 PMC3441783 22726453

[61] Sardinha LB, Marques A, Minderico C, Ekelund U. Cross-sectional and prospective impact of reallocating sedentary time to physical activity on children’s body composition. Pediatr Obes. 2017;12. 10.1111/ijpo.12153 PMC6258907 27256488

[62] Ayala AMC, Sudholz B, Salmon J, Dunstan DW, Ridgers ND, Arundell L et al. The impact of height-adjustable desks and prompts to break-up classroom sitting on adolescents’ energy expenditure, adiposity markers and perceived musculoskeletal discomfort. PLoS ONE. 2018;13. 10.1371/journal.pone.0203938 PMC6147438 30235241

[63] Watson A, Timperio A, Brown H, Best K, Hesketh KD. Effect of classroom-based physical activity interventions on academic and physical activity outcomes: a systematic review and meta-analysis. Int J Behav Nutr Phys Act. 2017. 10.1186/s12966-017-0569-9 PMC5574081 28841890

[64] Salmon J, Arundell L, Cerin E, Ridgers ND, Hesketh KD, Daly RM et al. Transform-Us! Cluster RCT: 18-month and 30-month effects on children’s physical activity, sedentary time and cardiometabolic risk markers. Br J Sports Med. 2023;57. 10.1136/bjsports-2022-105825 PMC9985722 36428089

[65] Dunstan DW, Dogra S, Carter SE, Owen N. Sit less and move more for cardiovascular health: emerging insights and opportunities. Nat Rev Cardiol. 2021. 10.1038/s41569-021-00547-y 34017139

[66] Yang Y, Schumann M, Le S, Cheng S. Reliability and validity of a new accelerometer-based device for detecting physical activities and energy expenditure. PeerJ. 2018;2018. 10.7717/peerj.5775 PMC6186411 30324032

