# Supplementary material for: Charting the cascade of physical activities: implications for reducing sitting time and obesity in children
Source: J Act Sedentary Sleep Behav. 2024 Jun 13;3:14. doi: 10.1186/s44167-024-00053-9 (PMC11960387; doi:10.1186/s44167-024-00053-9)
Supplement: Supplementary file 1 — Supplementary Material 1 [file 44167_2024_53_MOESM1_ESM.docx]

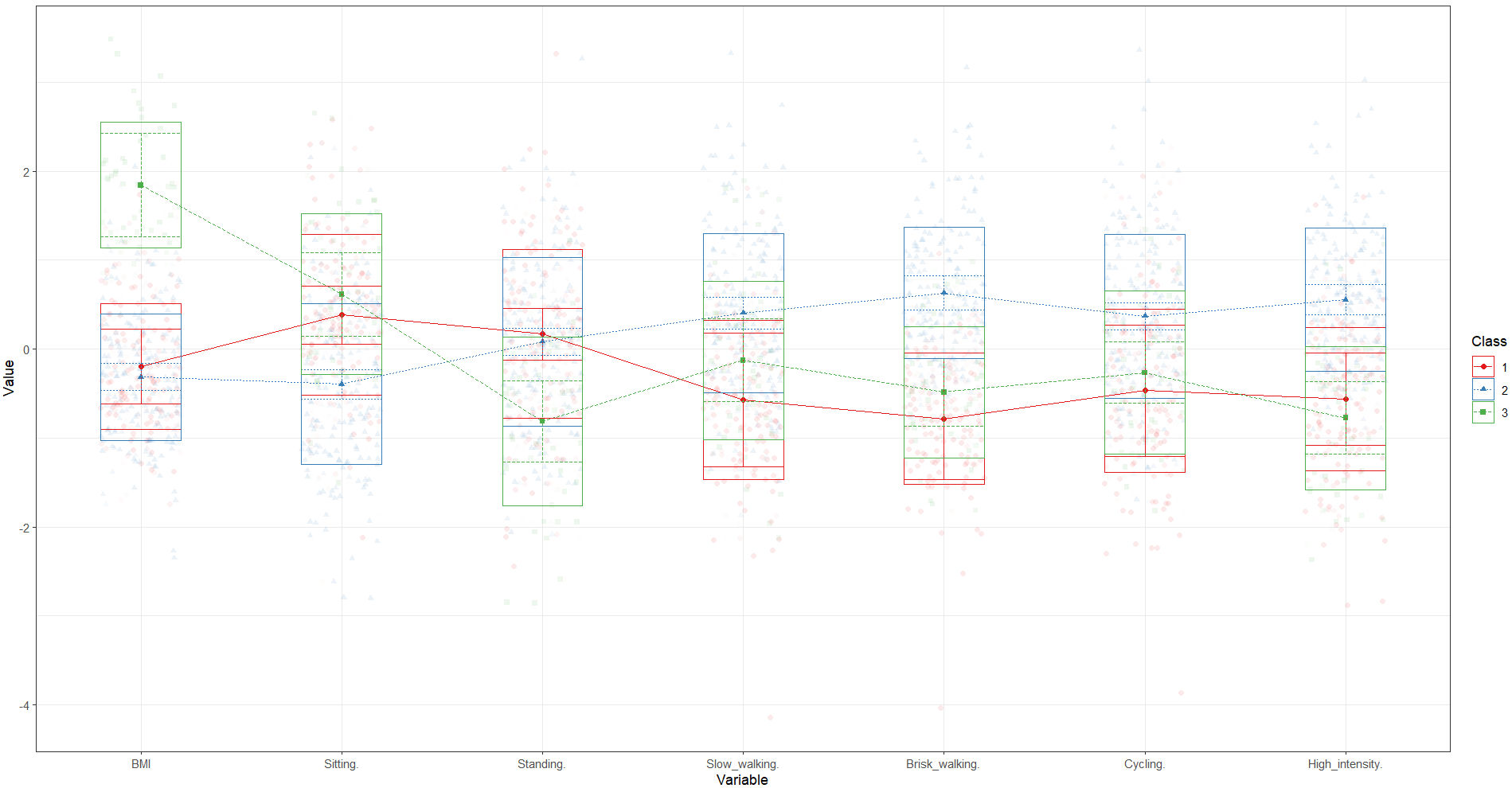


**Figure 1**. Standardized group averages on physical activity, sitting and BMI for three-profile solution.

[Class 1 = Normal BMI – high sitting – high standing – low slow-walking – low brisk-walking – low cycling – low high-intensity]; [Class 2 = Normal BMI – low sitting – high standing – high slow-walking – high brisk-walking – high cycling – high high-intensity]; [Class 3 = High BMI – high sitting – low standing – low slow-walking – low brisk-walking – low cycling – low high-intensity]


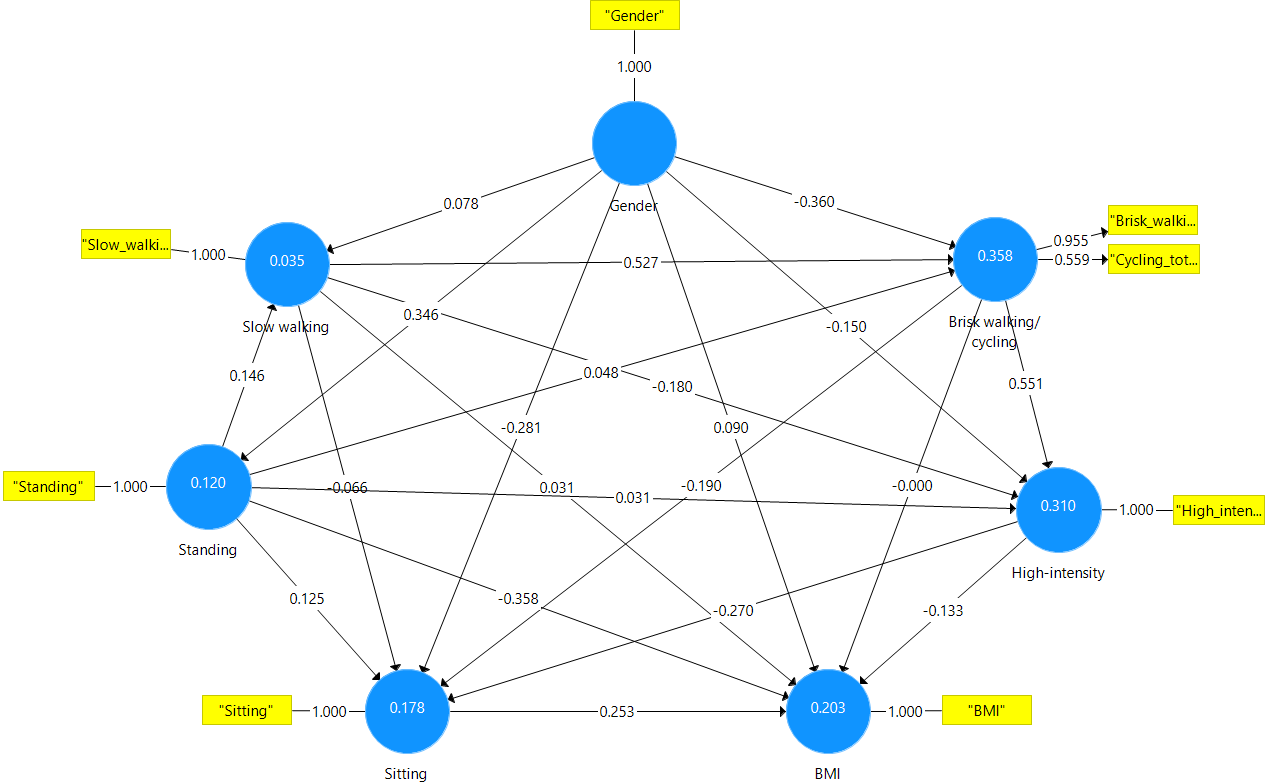


**Figure 2.** First construct of PLS**-**SEM predicting BMI in children after removing non-significant associations

**Table 1**. Total indirect effects among variables

|  | **Sample Mean** | **SD** | **T Statistics** | **Effect size** | ***p*** |
| --- | --- | --- | --- | --- | --- |
| "High-intensity -> BMI" | -0.057 | 0.021 | 2.802 | **0.0032** | 0.005 |
| "Brisk walking/cycling -> BMI" | -0.176 | 0.031 | 5.647 | **0.031** | <0.001 |
| "Brisk walking/cycling -> Sitting" | -0.142 | 0.042 | 3.418 | **0.020** | 0.001 |
| "Slow walking -> BMI" | -0.055 | 0.016 | 3.442 | **0.003** | 0.001 |
| "Slow walking -> High-intensity" | 0.296 | 0.033 | 8.944 | **0.088** | <0.001 |
| "Slow walking -> Sitting" | -0.155 | 0.033 | 4.716 | **0.024** | <0.001 |
| "Standing -> Brisk walking/cycling " | 0.091 | 0.03 | 3.022 | **0.008** | 0.003 |
| "Standing -> Sitting" | -0.026 | 0.01 | 2.662 | **0.0007** | 0.008 |

**Table** **2**. Specific indirect effects among variables

|  | Sample Mean | SD | T Statistics | P Values | *Effect size* |
| --- | --- | --- | --- | --- | --- |
| Brisk walking/Cycling -> High-intensity -> BMI | -0.09 | 0.03 | 3.44 | 0 | 0.0081 |
| Slow walking -> Brisk walking/Cycling -> High-intensity -> BMI | -0.05 | 0.01 | 3.51 | 0 | 0.0025 |
| Standing -> Slow walking -> Brisk walking/Cycling -> High-intensity -> BMI | -0.01 | 0 | 2.26 | 0.02 | 0.0001 |
| Slow walking -> High-intensity -> BMI | 0.03 | 0.01 | 2.59 | 0.01 | 0.0009 |
| Standing -> Slow walking -> High-intensity -> BMI | 0 | 0 | 2.07 | 0.04 | 0 |
| Brisk walking/Cycling -> Sitting -> BMI | -0.05 | 0.02 | 2.62 | 0.01 | 0.0025 |
| Slow walking -> Brisk walking/Cycling -> Sitting -> BMI | -0.03 | 0.01 | 2.58 | 0.01 | 0.0009 |
| Standing -> Slow walking -> Brisk walking/Cycling -> Sitting -> BMI | 0 | 0 | 2.07 | 0.04 | 0 |
| Brisk walking/Cycling -> High-intensity -> Sitting -> BMI | -0.03 | 0.01 | 2.53 | 0.01 | 0.0009 |
| Slow walking -> Brisk walking/Cycling -> High-intensity -> Sitting -> BMI | -0.02 | 0.01 | 2.62 | 0.01 | 0.0004 |
| Standing -> Slow walking -> Brisk walking/Cycling -> High-intensity -> Sitting -> BMI | 0 | 0 | 1.85 | 0.06 | 0 |
| High-intensity -> Sitting -> BMI | -0.06 | 0.02 | 2.72 | 0.01 | 0.0036 |
| Slow walking -> High-intensity -> Sitting -> BMI | 0.01 | 0.01 | 1.91 | 0.05 | 0.0001 |
| Standing -> Slow walking -> High-intensity -> Sitting -> BMI | 0 | 0 | 1.56 | 0.12 | 0 |
| Standing -> Sitting -> BMI | 0.03 | 0.01 | 1.82 | 0.05 | 0.0009 |
| Standing -> Slow walking -> Brisk walking/Cycling | 0.09 | 0.03 | 2.93 | 0 | 0.0081 |
| Slow walking -> Brisk walking/Cycling -> High-intensity | 0.3 | 0.03 | 9.07 | 0 | 0.9 |
| Standing -> Slow walking -> Brisk walking/Cycling -> High-intensity | 0.05 | 0.02 | 2.82 | 0 | 0.0025 |
| Standing -> Slow walking -> High-intensity | -0.03 | 0.01 | 2.19 | 0.03 | 0.0009 |
| Slow walking -> Brisk walking/Cycling -> Sitting | -0.12 | 0.04 | 3.46 | 0 | 0.0144 |
| Standing -> Slow walking -> Brisk walking/Cycling -> Sitting | -0.02 | 0.01 | 2.44 | 0.01 | 0.0004 |
| Brisk walking/Cycling -> High-intensity -> Sitting | -0.14 | 0.04 | 3.36 | 0 | 0.0196 |
| Slow walking -> Brisk walking/Cycling -> High-intensity -> Sitting | -0.08 | 0.02 | 3.42 | 0 | 0.0064 |
| Standing -> Slow walking -> Brisk walking/Cycling -> High-intensity -> Sitting | -0.01 | 0.01 | 2.16 | 0.03 | 0.0001 |
| Slow walking -> High-intensity -> Sitting | 0.05 | 0.02 | 2.23 | 0.03 | 0.0025 |
| Standing-> Slow walking -> High-intensity -> Sitting | 0.01 | 0 | 1.77 | 0.08 | 0.0001 |

Note: the table show the specific indirect effects of PLS-SEM results when including all variables in each association (i.e., results when existing or removing each variable)
